# Advanced-technique radiation therapy for nasopharyngeal carcinoma in a low resource setting: a review of treatment-related quality of life

**DOI:** 10.3332/ecancer.2024.1770

**Published:** 2024-09-18

**Authors:** Godwin Uwagba, Adedayo Joseph, Muhammed Habeebu, Eben Aje, Aishat Oladipo, Olufunmilayo Fagbemide, Precious Akowe, Azeezat Ajose, Adebayo Abe, Samuel Adeneye, Ibrahim Elhamamsi, Abdallah Kotkat, Nusirat Adedewe, Inioluwa Ariyo, Wonuola Adetugbogbo, Francis Durosinmi-Etti

**Affiliations:** 1 NSIA -LUTH Cancer Centre, Lagos University Teaching Hospital, Lagos 100001, Nigeria; 2 Department of Radiation Biology, Radiotherapy, and Radiodiagnosis, College of Medicine, University of Lagos, Lagos 100001, Nigeria; 3 Research Department, NSIA-LUTH Cancer Centre, Lagos University Teaching Hospital, Lagos 100001, Nigeria; 4 College of Medicine, University of Lagos, Lagos 100001, Nigeria

**Keywords:** nasopharyngeal carcinoma, health-related quality of life, advanced-technique radiation, IMRT, Nigeria

## Abstract

**Background:**

Nasopharyngeal carcinoma (NPC) is a rare but significant public health concern, especially in Africa, with a rising global incidence. This study aimed to investigate the pattern of presentation, treatment outcomes and impact on health-related quality of life (HRQOL) of NPC patients at a tertiary institution in Lagos, Nigeria.

**Methodology:**

A retrospective review of all nasopharyngeal cancer patients (*n* = 125) treated at a tertiary centre in Lagos, Nigeria, from May 2019 to 2022 was done. The European Organisation for Research and Treatment of Cancer (EORTC) H&N 35 questionnaire was used to assess HRQOL at 1-year post treatment and the data were analysed using a statistical package for the social sciences v26.0.

**Results:**

Among 125 patients, the mean age was 46.21 ± 17.82 years with 76% male. Comorbidities were reported in 34 patients (27.2%), smoking history in 18 patients (14.4%) and 50 patients (40%) reported alcohol consumption. Environmental risk factors were identified in six patients (4.8%). The most prevalent histology was squamous cell carcinoma (92.8%), and stage IV was the most common stage (42.4%). Chemoradiation was the primary treatment (63.2%), with intensity-modulated radiotherapy being the most utilised approach (51.2%). Among 125 patients, 51 completed the EORTC questionnaire. Weight loss, sticky saliva, dry mouth, difficulties in swallowing and problems with the sense of taste and smell were the most severe symptoms reported by patients. In the follow-up, 79.2% of patients were reached (50.4% alive, 28.8% deceased). Mortality was significantly associated with age >65 years, weight loss at presentation and consumption of grilled/smoked food.

**Conclusion:**

The study highlights key aspects of NPC in our region including the predominance in males, advanced disease stage at presentation and persistent symptoms post-treatment. Our findings point to the need for targeted initiatives to improve early detection and quality of life for nasopharyngeal patients in the country.

## Background

Nasopharyngeal carcinoma (NPC) has unique characteristics that distinguish it from other epithelial tumours in the head and neck region. According to the International Agency for Research on Cancer (IARC), 133,354 new cases are diagnosed annually, with 72% of cases diagnosed in men [1]. It is a relatively uncommon cancer that accounts for less than 5% of all cancer cases, but with significant morbidity and mortality. In 2020, the IARC recorded eighty thousand nasopharyngeal cancer-related deaths [1]. NPC has a unique geographical distribution, rare in Western countries but endemic in specific regions such as Southeast Asia, North Africa, the Middle East and the Arctic [2–4]. In Africa, it is the 22nd most diagnosed malignancy, with a 5-year prevalence of 22,761 cases across all ages [5]. Historically, Martinson and Aghadiuno [6] documented an increasing incidence of NPC in Nigeria; attributed to a penetrance/pervasiveness of viral infections implicated in its pathogenesis in the region. However, it is difficult to accurately predict the recent national burden because of the predominance of hospital-based studies [7].

The primary cause of NPC is believed to be linked to genetic susceptibility and viral infections with the Epstein-Barr virus (EBV) and, on a much smaller scale, the human papilloma virus [2, 3] EBV which has been linked to NPC has been reported to be an endemic virus in Nigeria [6, 8]. Various environmental and lifestyle factors also contribute to the risk of developing NPC. These include consumption of certain preserved foods, salted fish and foods, alcohol, smoking habits and exposure to certain types of occupational dust and fumes. The interplay between genetic predisposition, viral infection and environmental factors is complex and needs to be fully understood [3, 9]. Frequently, NPC is diagnosed at advanced stages because of its anatomical location and nonspecific early symptoms [10]. In particular, patients in Sub-Saharan Africa present with advanced-stage disease as a result of a combination of non-specific symptoms and other healthcare infrastructural and workforce deficiencies [11–14]. Stage at presentation is a crucial determinant for treatment-related quality of life specifically during radiotherapy and, ultimately, survival outcomes [15, 16].

Radiotherapy is the primary treatment modality for NPC, with intensity-modulated radiotherapy (IMRT) being the preferred technique based on data showing that it reduces toxicity and improves target coverage by ensuring precise targeting while minimizing doses to surrounding normal tissue [17, 18]. Chemotherapy, as a neo-adjuvant, concurrent or adjuvant treatment, has been shown to improve regional control and overall survival outcomes in patients compared to the historical use of chemotherapy only in late-stage and metastatic diseases [19–21].

Nasopharyngeal cancer substantially impacts patients’ health-related quality of life (HRQOL) due to its distinctive anatomical location, resulting in symptoms and treatment-related side effects [22]. The location of the nasopharynx adjacent to essential structures in the head and neck region (brain, ears, salivary glands and optic nerve) puts these structures at risk of disease extension/infiltration and normal tissue damage from inclusion in the radiotherapy field. Long-term adverse effects such as hearing loss, speech defects, difficulty swallowing, xerostomia (dry mouth) and cognitive changes, which may persist and affect day-to-day life are common with NPC treatment [23]. Literature shows that treatment-related adverse effects such as dysphagia, dry mouth and mouth-opening difficulties profoundly impact the quality of life negatively [24, 25]. In recent times, advanced treatment techniques and the possibility of re-planning during treatment based on repeated imaging are beneficial to patients’ overall quality of life and survival [25, 26]. Understanding this impact is crucial for healthcare professionals to provide comprehensive care and support to patients affected by the disease. This study aimed to investigate the treatment outcomes and impact on HRQOL, among patients with NPC at a tertiary institution in Lagos, Nigeria.

## Methods

### Study design and patient selection

A retrospective analysis was conducted on 125 adult patients with histologically diagnosed NPC at the NSIA-LUTH Cancer Center, Lagos University Teaching Hospital (LUTH), Nigeria, over 3 years starting from May 2019 to 2022. Data on sociodemographic characteristics, clinical history, treatment modalities and radiotherapy dose details, HRQOL documented at 1-year post treatment follow up visit were obtained from clinical case files and patients’ electronic medical records. Verbal informed consent was obtained from participants before follow-up. Ethical consideration was obtained from the LUTH Health Research and Ethics Committee. Patient information was anonymized and assigned unique identifiers prior to statistical analysis.

### Treatment

According to the cancer staging manual by the American Joint Committee on Cancer, eighth edition, disease staging was categorised by location and Tumour, Node, and Metastasis (TNM) criteria to stages I–IVA and IVB. The treatment protocol at our institution, following current guidelines, involves conformal radiotherapy (IMRT or volumetric modulated arc therapy (VMAT)) for all localised and locally advanced NPC, with chemotherapy (neo-adjuvant, concurrent and adjuvant) administered as indicated per National Comprehensive Cancer Network guidelines. Patients with disseminated metastatic disease receive systemic treatment only or best supportive care based on performance status.

### Quality of life instrument

The European Organisation for Research and Treatment of Cancer (EORTC) H&N 35 questionnaire was used to assess these patients’ HRQOL [27]. This is a validated tool used to measure the impact of head and neck cancers on patients’ quality of life from their perspective. The QLQ-H&N35 comprises seven multi-item scales that assess pain, swallowing, taste and smell senses, speech difficulties, social eating, social contact and physical appearance. Furthermore, eleven single items survey problems with teeth, mouth opening, dry mouth, sticky saliva, coughing, feeling ill, use of pain medications, nutritional supplements or a nasogastric tube and weight changes (gain or loss) [27]. In this study, QLQ-H&N35 was administered to patients by an interviewer within a 2-week duration while assessing survival outcomes.

### Follow-up

After the completion of treatment, patients were followed up with a routine clinical consultation, physical examination and imaging of the head and neck (nasopharynx) and other body parts as indicated. Our centre routinely collects data during patient visits at different time points for patients with head and neck cancers, including NPC to guide treatment. For this study, we report on the HRQOL data collected at the 1-year post treatment date for our nasopharyngeal cancer patients. This data were gathered either in-person during the patient’s scheduled follow-up visit or via telephone within 2 weeks of the 1-year follow-up date by trained centre staff, ensuring all patients were assessed within a consistent timeframe. Each patient’s 1-year follow-up point was personalised based on the date of their completion of radiation therapy. Thus, the 1-year assessments occurred on different dates specific to each patient’s treatment timeline. Verbal consents were obtained before each consultation. Loss to follow-up was determined as any patient who missed three consecutive follow-up appointments and was unreachable via other provided forms of communication, in which case survival status was determined by records from the last clinic visit.

### Statistical analysis

THE EORTC H&N35 scoring manual was used to calculate the score to range from 0 to 100 [27]. Data were analysed using a statistical package for the social sciences 26.0 statistics software. Data were presented in frequencies, percentages, tables and means with standard deviations. Mann–Whitney *U* and Kruskal–Wallis tests were used to examine associations between different quantitative variables and the significance level was set at *p* < 0.05.

## Results

One hundred and twenty-five patients, with a mean age of 46.21 ± 17.82 years (range, 9–97) were treated for NPC in the study institution within the 3-year duration. The predominant gender was male (76%). Patient characteristics are summarized in Table 1.

Prevalent comorbidities were reported in 34 patients (27.2%). Of these, 26 patients had hypertension alone, 7 had co-existing hypertension and type 2 diabetes mellitus and 1 had type 2 diabetes mellitus. Smoking history was present in 18 patients (14.4%) and 50 patients (40%) reported alcohol consumption while 14.4% reported both alcohol consumption and smoking. Environmental risk factors were identified in six patients (4.8%). The most prevalent histology was squamous cell carcinoma (92.8%) and stage IV was the most common stage (Table 2). Overall, following institutional treatment protocols, 15 patients (12%) had a prior surgical intervention, 79 patients (63.2%) were treated with chemoradiation and 29 patients (23.2%) had radiotherapy alone, with IMRT being the most utilised approach (51.2%) and VMAT was used for 41.6%. A total of 15 patients (12.0%) in this study population have had surgery. Of these, 14 (11.2%) had chemoradiation in addition to the surgical procedure, while only 1 (0.8%) had chemotherapy together with surgery.

Among the 125 patients, 51 completed the EORTC questionnaire (Table 3). In the H&N-35 module, weight loss, sticky saliva, dry mouth, difficulties in swallowing and problems with the sense of taste and smell were the most severe symptoms reported by patients. In the follow-up, 99 patients were reached (63 alive, 36 deceased). Mortality at 1 year was significantly associated with age >65 years, weight loss at presentation and dietary consumption of grilled/smoked food (Table 4).

## Discussion

This article describes the clinicopathological characteristics, treatment outcomes and quality of life at 1-year post treatment of patients with nasopharyngeal cancer in Lagos, Nigeria. The male-to-female ratio in this study was 3.2:1. This correlates with several studies from all over the world, including African countries, which show that NPC predominantly affects the male gender [15, 22, 28–30]. Most patients (43.2%) in this study were 45–64 years old. Age group distribution of NPC typically varies between endemic and non-endemic regions. In a tertiary hospital in Indonesia, most patients diagnosed were within the 41–50 age group [30]. Also, in the Asian high-risk population, the incidence of NPC peaks between ages 45–59 years. Meanwhile, it is mainly found among the young adult population in non-endemic regions [3]. The clinical presentation of NPC largely depends on the extent of the disease spread anatomically. Thus, early stage diseases usually present with non-specific symptoms leading to missed and delayed diagnoses [2, 4, 31]. All patients in this study had initially presented at other centres. Thus, at a presentation at our institution, they had typical symptoms of advanced disease spread, such as epistaxis, nasal blockage, neck swelling (nodal involvement), hearing impairment (cranial nerve deficit), recurrent upper respiratory tract infection (URTI), headache, weight loss, dysphagia, visual impairment, hoarseness and mouth swelling, with epistaxis having the highest frequency (56%). This corresponds with other Nigerian studies which showed similar clinical presentations at tertiary institutions [28, 29].

Tobacco smoking, alcohol consumption and consumption of grilled foods were the common potential risk factors identified among this study population, with environmental exposure to toxic chemicals being the least (4.8%). We identified exposure to potential environmental toxins in six patients; some of the toxins include construction materials, metal and wood dust, motor fuels, acetone, bromophol fumes and paint products. In this study, a vast majority of the patients presented with advanced disease stages, that is, stages III (32%) and IV (42.4%),

respectively, while the most common histology finding was squamous cell carcinoma (92.8%). The late stage at diagnosis is a relatively common feature of NPC in both endemic and non-endemic regions because of its non-specific presentation in the early stages [2, 4]. Similar to the findings in this study, Cengiz *et al* [23] also observed that 75% of cases in his study population presented with an advanced stage. Also, 54% of the patients in a single institution study in Indonesia presented with stage IV disease [30]. With these similar figures from different parts of the world, the survival outcome rates would be expected to follow the same trend, but that is not the case. The difference in healthcare infrastructures and the availability of quality cancer care have been postulated to be the drivers for higher mortality rates in low and middle-income countries.

According to the World Health Organisation, NPC is classified into three histological subtypes: Keratinizing squamous cell carcinoma, non-keratinizing differentiated and undifferentiated NPC [32]. From the histological classification of patients in this study, 36.0% had been diagnosed with undifferentiated NPC. This finding, along with similar observations of predominantly NPC cases of the non-keratinizing histology type in other centres across Nigeria (Ibadan, Ilorin and Kaduna), further proves that an increase in NPC burden can be linked to EBV infections [28, 29]. All 15 patients who underwent surgery had previous interventions outside our centre, which primarily offers chemotherapy and radiotherapy services. Specifically, three patients had elective tracheostomies, seven patients had endoscopic sinus surgery aimed at tumour excision, and one patient had undergone nasal polypectomies before the presentation. Additionally, four patients had a history of various other surgeries, including herniorrhaphy orchidectomy and neck surgery.

Radiotherapy treatment of NPC requires a wide treatment field that may affect several vital structures such as the parotid gland, muscles of mastication, temporomandibular joint and other neural structures [23, 33]. Advancements in cancer treatment modalities have improved survival outcomes for NPC patients. However, the increase in long-term survival has led to a higher burden of long-term toxicities that impact patients’ quality of life. In our study, the findings from the EORTC QLQ-H&N35 questionnaire showed that weight loss, sticky saliva, dry mouth, difficulties in swallowing and problems with the sense of taste and smell were the most severe symptoms reported by patients [25]. These symptoms are primarily related to xerostomia, a condition frequently cited as one of the most prevalent complications following radiotherapy for head and neck cancers. Our results align closely with those reported by Fang *et al* [25], who also identified dry mouth and sticky saliva as predominant symptoms in their cohort. Similarly, Huang *et al* [33], in a longitudinal study, observed that symptoms related to swallowing, taste, mouth opening, dry mouth and sticky saliva remained significantly worse at 1-year post-treatment in patients with nasopharyngeal cancer. Our findings suggest that the impacts of NPC and its treatment on quality of life are profound and persist long after the initial treatment period has ended. Our study shows a need to integrate supportive care and symptom management for NPC patients during and after treatment. Additionally, there should be an emphasis on nutritional support and counseling, as weight loss and swallowing difficulties can lead to significant nutritional deficiencies, further impacting the patient’s recovery and quality of life.

79.2% (99) of patients had follow-up calls and 20.8% (26) were lost to follow-up. Of the 99 patients who were reached, 50.4% were alive and the remaining 28.8% were reported dead. Mortality was significantly associated with age >65, weight loss at presentation and dietary consumption of grilled/smoked food. Our limitations include a primary focus on the quality of life assessments at the 1-year post-treatment mark, with only a preliminary overview of overall survival rates provided. Detailed analyses of disease control and progression-free survival were not included in this study. The focus of our next research will be to extend our follow-up with these patients, aiming to report on these critical outcomes up to 5 years post-treatment. This expanded study will provide a comprehensive view of long-term survival and disease control, which will be invaluable for understanding outcomes for NPC in low-resource settings. Additionally, while our current study did not investigate the impact of local habits or genetic factors, these represent significant variables that could influence outcomes. Future studies should explore how local habits and genetic predispositions may uniquely affect our patient population.

## Conclusion

Despite advancements in radiotherapy that have improved survival rates, NPC continues to present significant challenges due to late-stage diagnosis and the resultant severe long-term toxicities that adversely affect quality of life. Our findings reveal that symptoms such as weight loss, sticky saliva, dry mouth and swallowing difficulties are prevalent and persist long after treatment and this shows the need for integrated supportive care and comprehensive symptom management for these patients during and after treatment. Long-term follow-up important to document the effects of treatment on quality of life and survival and allow clinicians to identify interventions that mitigate long-term toxicities and improve overall patient quality of life and survival outcomes.

## List of abbreviations

EBV, Epstein-Barr virus; EORTC, European Organization for Research and Treatment of Cancer; H&N, Head and neck; HRQOL, Health-related quality of life; IMRT, Intensity-modulated radiation therapy; NPC, Nasopharyngeal carcinoma; NSIA-LUTH, Nigerian Sovereign Investment Authority-Lagos University Teaching Hospital; URTI, Upper respiratory tract infection; VMAT, Volumetric modulated arc therapy.

## Conflicts of interest

The authors declare that there are no conflicts of interest regarding the publication of this manuscript; and no other relationships or activities that could appear to have influenced the submitted work.

## Author contributions

All authors contributed to the conception and design of the study.

Godwin Uwagba, Adedayo Joseph, Olufumilayo Fagbemide and Azeezat Ajose contributed to the data analysis of the work.

All authors contributed to the drafting and revision of the manuscript.

All authors approved the final version for publication.

All authors agree to be accountable for all aspects of the work.

Adedayo Joseph is the corresponding author and provided overall leadership for all aspects of the study.

## Data sharing statement

Supporting information is available from the corresponding author on request.

## Figures and Tables

**Table 1.** Sociodemographic and patient characteristics.

Variable	Frequency (*n*)	Percentage (%)
Gender		
Male	95	76.00
Female	30	24.00
Age group		
<18	4	3.20
18–24	15	12.00
25–44	36	28.80
45–64	54	43.20
> 65	16	12.80
Marital status		
Single	35	28.00
Married	86	68.80
Separated	1	0.80
Widowed	3	2.40
Monthly income (US$ 1–500 NGN)		
<$100	31	24.80
$100–$300	45	36.00
$300–$500	33	26.40
> $500	16	12.80
Clinical presentation		
Epistaxis	70	56.00
Nasal blockage	59	47.20
Neck swelling	50	40.00
Hearing impairment	43	34.40
Recurrent URTI	41	32.80
Headache	40	32.00
Weight loss	31	24.80
Dysphagia	24	19.00
Visual impairment	20	16.00
Hoarseness	14	11.20
Mouth swelling	6	4.80
Risk factors		
Smoking	18	14.40
Alcohol consumption	50	40.00
Smoking and alcohol	18	14.40
Environmental	6	4.80
Grilled foods	25	20.00
Family history of cancer	9	7.20
Comorbidities		
Present	34	27.20
Absent	91	72.80

**Table 2.** Tumour and treatment characteristics.

S	Frequency (*n*)	Percentage (%)
Stage at presentation		
I	5	4.00
II	24	19.20
III	40	32.00
IV	53	42.40
Unstaged	3	2.40
Histopathology		
Squamous cell carcinoma	116	92.80
Lymphoepithelioma carcinoma	4	3.20
B-cell lymphoma	1	0.80
Adenocarcinoma	1	0.80
Others	3	2.40
Treatment		
Radiotherapy	29	23.20
Chemoradiation	79	63.20
Chemotherapy	2	1.60
Surgery + chemoradiation	14	11.20
Surgery + chemotherapy	1	0.80
Radiotherapy technique		
IMRT	64	51.20
VMAT	52	41.60
Outcome		
Alive	63	50.40
Dead	36	28.80
Lost to follow-up	26	20.80

**Table 3.** HRQOL scores (QLQ-H&N-35).

Symptoms	*N*	Minimum	Maximum	Mean	SD
Pain	51	0.00	91.67	24.1830	25.18018
Swallowing	51	0.00	100.00	30.8824	27.95304
Sense problems	51	0.00	100.00	24.5098	27.35628
Speech difficulties	51	0.00	88.89	20.9150	24.50972
Social eating	51	0.00	100.00	20.2070	24.74611
Social contact	51	0.00	100.00	19.6078	27.37109
Less sexuality	51	0.00	66.67	14.7059	24.86565
Teeth	51	0.00	66.67	7.8431	19.53713
Mouth opening	51	0.00	100.00	16.9935	28.57547
Dry mouth	51	0.00	100.00	32.0261	36.49096
Sticky saliva	51	0.00	100.00	33.3333	34.64102
Coughing	51	0.00	100.00	13.7255	25.10110
Feeling ill	51	0.00	100.00	10.4575	23.56098
Pain medications	51	0.00	100.00	39.3157	49.30895
Nutritional supplements	51	0.00	100.00	47.0588	50.41008
Feeding tubes	51	0.00	100.00	7.8431	27.15244
Weight loss	51	0.00	100.00	56.8627	50.01960
Weight gain	51	0.00	100.00	9.8039	30.03266

**Table 4.** Factors associated with poor outcomes among NPC patients (*n* = 99).

Variable	Alive *N* = 63	Dead *N* = 36	*X* ^2^	*p*-value
Age				
<65 years	58 (69.05%)	26 (30.95%)	7.015	0.017
>65years	5 (33.33%)	10 (66.67%)		
Gender				
Female	18 (75%)	6 (25%)	1.768	0.228
Male	45 (60%)	30 (40%)		
Marital status				
Married	42 (60.87%)	27 (39.13%)	0.753	0.496
Not married	21 (70%)	9 (30%)		
Estimated monthly income				
<$300	40 (63.49%)	23 (36.51%)	0.002	1
>$300	23 (63.89%)	13 (36.11%)		
Smoking history				
Yes	55 (63.95%)	31 (36.05%)	0.028	1
No	8 (61.54%)	5 (38.46%)		
Alcohol history				
Yes	41 (69.49%)	18 (30.51%)	2.163	0.201
No	22 (55%)	18 (45%)		
Comorbidity				
Yes	16 (55.17%)	13 (44.83%)	1.270	0.359
No	47 (67.14%)	23 (32.86%)		
Consumption of grilled food				
Yes	5 (22.73%)	17 (77.27%)	20.457	<0.001
No	58 (75.32%)	19 (24.68%)		
Weight loss at presentation				
Yes	9 (31.03%)	20 (68.97%)	18.839	0
No	54 (77.14%)	16 (22.86%)		
Stage at presentation				
Localised disease	22 (88%)	3 (12%)	8.579	0.004
Advanced	41 (55.41%)	33 (44.59%)		
